# Nanoscale assembly of superconducting vortices with scanning tunnelling microscope tip

**DOI:** 10.1038/ncomms13880

**Published:** 2016-12-09

**Authors:** Jun-Yi Ge, Vladimir N. Gladilin, Jacques Tempere, Cun Xue, Jozef T. Devreese, Joris Van de Vondel, Youhe Zhou, Victor V. Moshchalkov

**Affiliations:** 1 Department of Physics and Astronomy, INPAC–Institute for Nanoscale Physics and Chemistry, KU Leuven, Celestijnenlaan 200D, B-3001 Leuven, Belgium; 2 TQC–Theory of Quantum and Complex Systems, Department of Physics, Universiteit Antwerpen, Universiteitsplein 1, B-2610 Antwerpen, Belgium; 3 School of Mechanics, Civil Engineering and Architecture, Northwestern Polytechnical University, Xi'an 710071, China; 4 School of Aeronautics, Northwestern Polytechnical University, Xi'an 710071, P.R. China

## Abstract

Vortices play a crucial role in determining the properties of superconductors as well as their applications. Therefore, characterization and manipulation of vortices, especially at the single-vortex level, is of great importance. Among many techniques to study single vortices, scanning tunnelling microscopy (STM) stands out as a powerful tool, due to its ability to detect the local electronic states and high spatial resolution. However, local control of superconductivity as well as the manipulation of individual vortices with the STM tip is still lacking. Here we report a new function of the STM, namely to control the local pinning in a superconductor through the heating effect. Such effect allows us to quench the superconducting state at nanoscale, and leads to the growth of vortex clusters whose size can be controlled by the bias voltage. We also demonstrate the use of an STM tip to assemble single-quantum vortices into desired nanoscale configurations.

## Abstract (web summary)

Scanning tunneling microscope (STM) is a powerful tool but local control of superconductivity with the STM tip is still lacking. Here, Ge *et al*. show the use of an STM tip to control the local pinning in a superconductor through the heating effect, allowing to manipulate single superconducting vortex at nanoscale.

Pair condensation of charge carriers from the normal to the superconducting state is associated with a gain of the free energy. However, in the presence of a magnetic field, this gain is decreased due to the expulsion of the external field by the superconductor. For type-II superconductors, this competition results in the penetration of quantized vortices which are characterized by two length scales: the coherence length *ξ*, equivalent to the size of the vortex core, and the magnetic penetration depth *λ*, the decay length for the supercurrents encircling a vortex core^1^. Since the formation of the vortex core requires breaking of Cooper pairs, it is energetically favourable for a vortex to be placed in an area where superconductivity is already locally suppressed. Such positions are called pinning centres and have various forms such as atom vacancies^2^, variations of sample composition or/and thickness^3^ and artificially patterned antidots^4^,^5^. The manipulation and control of pinning configuration and its interaction with vortex are fundamental for creating new functional devices with superconductors^6^,^7^,^8^.

The scanning tunnelling microscope (STM) is one of the most powerful techniques to image nanoscale topography and characterize the local physical properties^9^,^10^,^11^,^12^. It has been widely used in the study of condensed matter systems such as insulators^13^, semiconductors^14^ and superconductors^15^. Also the capability of precise positioning makes it a powerful tool to design various artificial nanostructures at atomic level^16^,^17^,^18^,^19^. In the study of superconductivity, the STM can probe the local electronic density of states and thus is used to map the spatial distribution of superconducting gap and the vortex core state. The STM is the most suitable technique to directly image vortices with atomic resolution, especially at high magnetic fields. A series of intricate phenomena related to vortex states have been revealed by the STM, such as the vortex lattice melting process^20^, the anisotropy of the Fermi surface distribution of the superconducting gap^21^, order–disorder transition^22^ to name a few. However, despite the importance of technical and scientific applications by utilizing vortices to design fluxonic devices^23^,^24^, local control of superconductivity and the precise manipulation of vortices with the STM is still lacking.

Here, we report a method of controlled quenching of a hot spot in a superconducting film by using the local heating effect of the tunnelling junction on vortex states, which is especially interesting at the nanoscale. This method utilizes the heating effect of the tunnelling junction with the power which can be well controlled by tuning the bias voltage. We are also able to use this technique to precisely manipulate single-quantum vortices. Our results extend the pioneering work of Eigler and Schweizer^16^ from manipulating single atoms to manipulating single-quantum vortices.

## Results

### Sample design and vortex assembling

**Figure 1:**
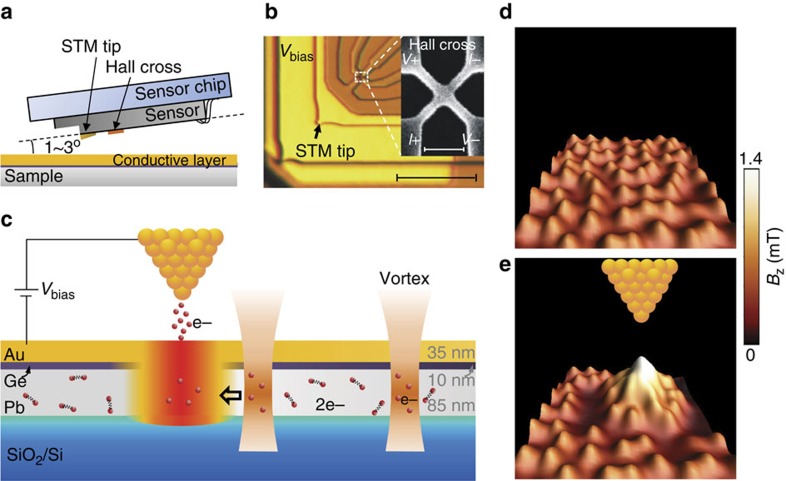
Introduction to the operation of the heating effect generated with a scanning tunnelling microscope tip. (**a**) Schematic view of the scanning Hall probe microscopy (SHPM). A scanning tunnelling microscope (STM) tip is assembled together with a Hall cross to make a sensor, which is aligned at a small angle (1–3°) to the sample surface. (**b**) An optical image of the Hall sensor. A scanning electron microscope (SEM) image of the Hall cross is shown in the inset. The longer (shorter) scale bar corresponds to 20 (1) μm. (**c**) Schematic representation of the local heating effect by using the STM tip. The area close to the tip is heated up by the tunnelling current while the insulating Ge layer and the superconducting Pb layer are also warmed up due to thermal transfer. Superconductivity is suppressed in a localized region where it is energetically favourable to place vortices. (**d**) SHPM image of a vortex lattice observed after FC at *H*=3.74 × 10^2^ A m^−1^ from above *T*_c_ down to *T*=4.2 K. (**e**) SHPM image after 5 s of tunnelling at bias voltage of 0.5 V and tunnelling current of 0.5 nA, and then lifting up the tip for Hall probe imaging. A vortex cluster forms at the tip position due to the local quench of the hot spot.

The experiments were performed by imaging vortices using scanning Hall probe microscopy (SHPM) which combines the non-invasive Hall imaging technique and an STM (Fig. 1a,b). The STM tip, typically used only to provide feedback to bring the Hall sensor in close proximity to the sample surface^25^, is here used as a local heating source. In the presence of tunnelling current, a pinning potential is created at the position of the tip due to the local suppression of the superconductivity. As a result, nearby vortices will be attracted to this region. However, once the tunnelling current disappears by retracting the tip to a certain distance (200 nm), the heating effect is stopped and the accumulated vortices will finally relax into a triangular vortex lattice due to the repulsive interaction among them. To avoid this and to retain the topological defects after quenching, a sample with sufficiently strong pinning centres and weak vortex–vortex interactions is required.

The sample schematically shown in Fig. 1c is a multilayered structure of Pb/Ge/Au deposited on a SiO_2_/Si substrate (see ‘Methods' section). The superconducting Pb film, which is characterized by relatively weak vortex–vortex interactions and a high density of quasi-homogeneously distributed pinning centres, is an ideal candidate to study the local heating effect. The pinning centres are created naturally during the sample preparation and can be considered as locations with a reduced electron mean free path^26^. The vortex–vortex interaction can be weakened by choosing a superconducting film with relatively small Ginzburg–Landau parameter *κ*=*λ*/*ξ*. Therefore, a Pb film with thickness *d*=85 nm, *λ*(0)=94 nm and *ξ*(0)=52 nm is used (Supplementary Figs 1–3, Supplementary Notes 1 and 2). In this case, once vortices are forced together, the attraction between pinning centres and vortices overcomes the repulsive interaction between vortices, so that a specific vortex configuration, formed in the course of fast hot-spot quenching, can be preserved.

Figure 1d presents the vortex distribution SHPM image after performing field cooling (FC) from above *T*_c_ to 4.2 K. A disordered vortex lattice, homogeneously distributed in the scanned area, is observed. After applying a pulse of tunnelling voltage, followed by the Hall probe imaging, a vortex cluster is observed at the STM tip position. Since there is no multi-quantum vortex observed in our sample, we assume that the vortex cluster is composed of single-quantum vortices (Supplementary Note 4). Between the vortex cluster and the rest of individual vortices a vortex-free area is formed. Note that the positions of the vortices outside the cluster remain unchanged. This confirms that the impact of heating is local. The vortex cluster retains the same structure even after a few hours of relaxation, suggesting that it is a relatively stable configuration. Similar results have been observed in three different samples. It has been reported that the STM tunnelling junction can reshape the topography of a material, generating defects at the tip position^27^. This possibility is ruled out since a disordered lattice is again observed in the same region after a subsequent FC procedure. The possibility of mechanically induced vortex attraction^28^ is also ruled out since, in our case, there is no direct contact between the STM tip and the sample.

### Controlling the vortex cluster size

**Figure 2:**
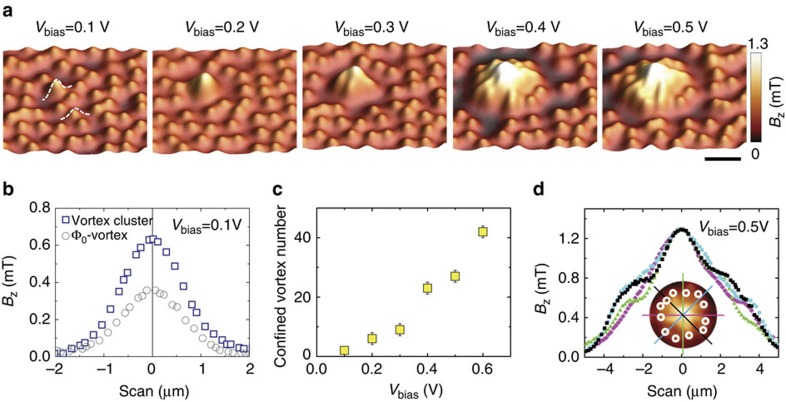
Vortex clustering as a function of bias voltage. (**a**) SHPM images observed after tunnelling pulses with various bias voltages *V*_bias_. The size of the vortex cluster increases with bias voltage. The minimum cluster that could be observed contains two flux quanta at *V*_bias_=0.1 V. The scale bar equals 4 μm. (**b**) Field profiles across the centre of an individual vortex (circles) and the smallest vortex cluster (squares), as indicated by the dashed lines in **a**, observed at *V*_bias_=0.1 V. (**c**) Number of vortices in the cluster as a function of the bias voltage (Supplementary Fig. 4, Supplementary Note 3). The error bar corresponds to the number of vortices cut by the edges of the scanned SHPM images. (**d**) Magnetic field profiles along different directions across the centre of a vortex cluster observed at *V*_bias_=0.5 V. All the profiles are similar to each other, suggesting that the vortex distribution in the cluster is approximately axially symmetric. The white circles mark the position of individual vortices at the periphery of the cluster (Supplementary Fig. 5).

The size of the vortex clusters can be well controlled. Figure 2a shows a series of images measured after applying the pulse of tunnelling current at the same position under various bias voltages. It is clear that the size of the vortex cluster increases with increasing bias voltage. The smallest vortex cluster is observed at *V*_bias_=0.1 V, below which no vortex clustering can be seen. Figure 2b compares the magnetic field profiles through the centres of the vortex cluster observed at *V*_bias_=0.1 V and a single-quantum vortex. It is seen that the magnetic field at the centre of the cluster is doubled as compared with a single-quantum vortex, suggesting that it contains two flux quanta (Supplementary Note 4). The number of trapped vortices as a function of the bias voltage is shown in Fig. 2c. The power dissipated in the sample is *P*∝*V*_bias_*I*, with *I* being the tunnelling current which is kept constant in our experiments. Correspondingly, the size of the region, where superconductivity is suppressed by local heating, is expected to increase with bias voltage. This accounts for the observed dependence in Fig. 2c.

One clear feature of the observed vortex clusters is that, instead of forming a disordered Abrikosov vortex lattice, clustered vortices are arranged into concentric ring-like structures. As demonstrated in Fig. 2d, the field profiles through the centre of a vortex cluster are nearly symmetric with a peak at the cluster centre. Such kind of ring-shaped vortex clusters, predicted by Shapiro *et al*.^29^ might be attributed to the Kibble–Zurek (KZ) symmetry-breaking phase transition^30^,^31^. When the tunnelling current is applied, a local area is heated up above *T*_c_, leading to the formation of a normal domain with homogeneous distribution of magnetic flux in this domain. After the heating is stopped by retracting the STM tip, the recovery of the superconductivity starts when the temperature front deviates from the order parameter front. The temperature front accelerates while the order parameter front, which presses the magnetic flux confined inside the shrinking normal region, decelerates, leading to an unstable normal domain ring area with *T*<*T*_c_ and 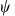
=0. As a result, vortices nucleate in this normal domain ring at the perimeter of the heated area and the hot spot looses part of its magnetic flux. The order parameter front then accelerates briefly, before the above-described dynamics repeat^29^,^32^. With further decreasing temperature, natural pinning in the heated area becomes evident again, and the ring-shaped vortex cluster is preserved even when the temperature of the whole area is far below *T*_c_. As far as we know, the ring-shaped vortex clusters have never been observed experimentally with single vortex resolution. The possible relation of our experiment to the KZ mechanism is further supported by the theoretical simulations, where vortex–antivortex pairs, as topological defects, appear from quenching the hot spot (Supplementary Figs 6–8, Supplementary Note 5). However, due to the short annihilation time, the stabilization of KZ vortices and antivortices have not been observed experimentally. Further experimental evidence is needed to clarify the mechanism, such as the relation of KZ vortices with quenching rate.

**Figure 3:**
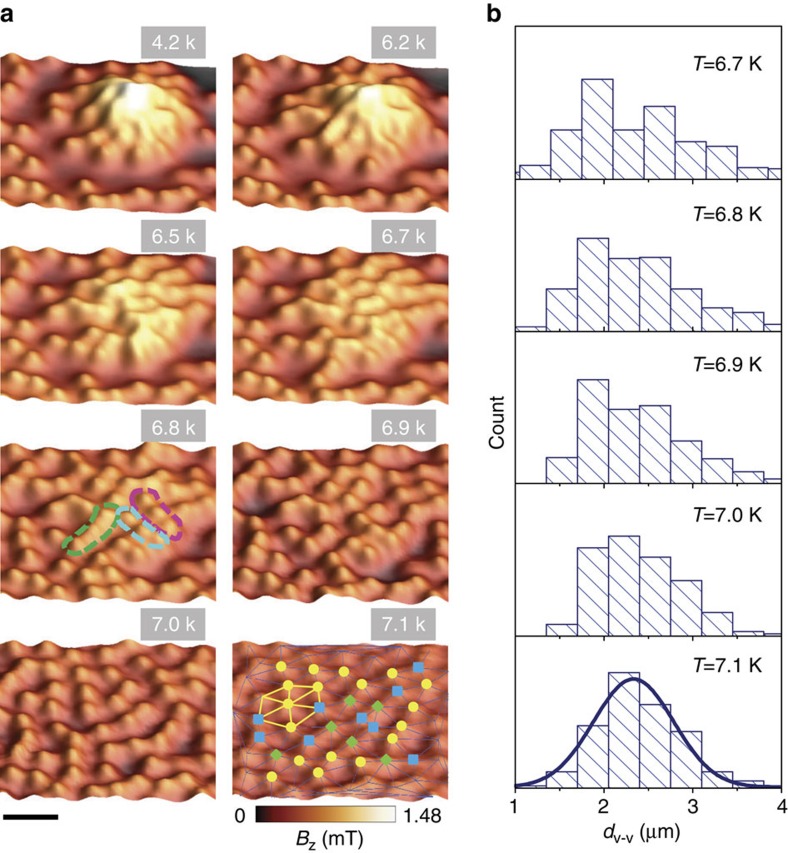
Disintegration of vortex cluster with increasing temperature. (**a**) SHPM images observed after forming a vortex cluster at 4.2 K and then increasing the temperature gradually. The vortex cluster remains compact until *T*=6.2 K. With further increasing temperature, the vortex cluster expands due to the weakening of pinning and finally a homogeneous vortex lattice is observed at *T*=7.1 K. The Delaunay triangulation of the vortex pattern at *T*=7.1 K is shown, where the vertices of each triangle represent the locations of the vortices. The squares (blue), circles (yellow) and diamonds (green) represent the vortices with five-, six- and seven-fold symmetries. The scale bar equals 4 μm. (**b**) Histograms of the vortex nearest neighbor distance (NND) obtained from the images in **a**. The distribution at 7.1 K can be well simulated using the Gaussian curve.

As mentioned above, the vortex clusters are preserved due to the naturally formed pinning centres. The pinning strength is weakened when approaching *T*_c_ (ref. 33). Therefore, we expect a competition between pinning and vortex lattice elasticity with varying temperature. This also provides an opportunity to study the relaxation of a local non-equilibrium configuration in a macroscopic system. Figure 3a displays the vortex cluster evolution with increasing temperature. When the vortex cluster is created at 4.2 K, it preserves the same geometry up to *T*=6.2 K (Supplementary Fig. 9), above which the pinning strength is considerably weakened as compared with the vortex–vortex interaction and the vortex cluster starts to disintegrate. We notice that the disintegration process starts from the interior of the vortex cluster, where the vortex density is high, so that the vortex–vortex interaction is strong. At intermediate temperatures (for example, *T*=6.8 K), vortices tend to form chains as highlighted by the dashed lines in Fig. 3a. Such vortex chains might be associated with the one-dimensional temperature-induced vortex motion as observed in the vortex lattice melting process^20^. Only at high enough temperature (for example, *T*=7.1 K), vortex repulsion strongly dominates and a homogeneous distribution of vortices, corresponding to a weakly distorted Abrikosov lattice, is formed. Additional information about the vortex cluster disintegration process can be obtained by considering the nearest neighbour distance (NND) of vortices. As presented in Fig. 3b, the NND shows a broad distribution at low temperatures. At *T*=7.1 K, it can be well fitted by the Gaussian distribution (solid line). The observed peak position at *d*_v–v_=2.3 μm is consistent with the value expected for an Abrikosov triangular vortex lattice, *d*_v–v_=(2Φ_0_/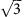
*H*_0_)^1/2^=2.27 μm.

### Theoretical simulations

**Figure 4:**
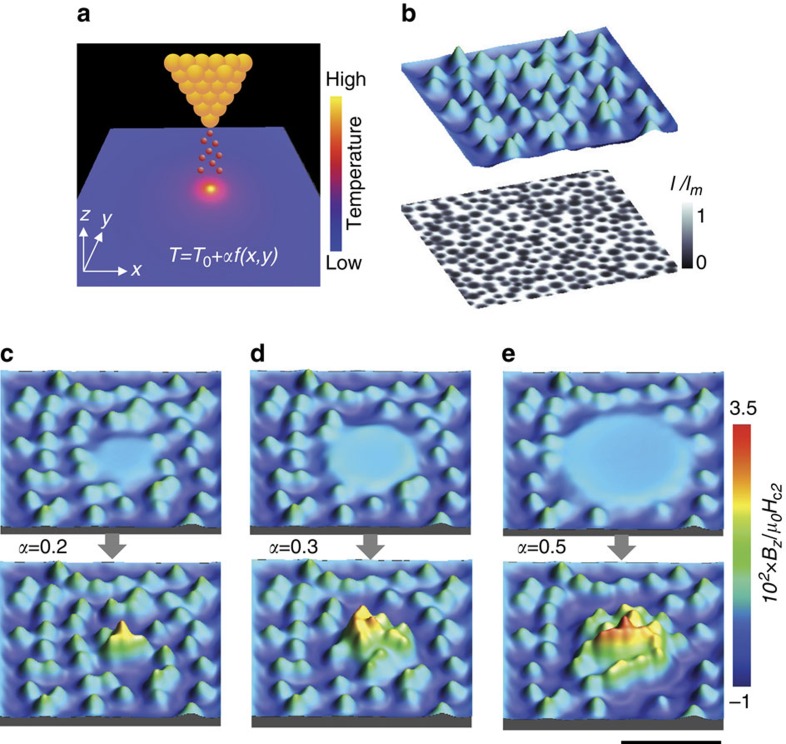
Simulations. (**a**) Temperature distribution at the scanning tunnelling microscope tip position. (**b**) Vortex distribution (upper panel) after FC down to the temperature *T*_0_=0.58 *T*_c_ at magnetic field *H*_0_=0.03*H*_c2_ (*T*_0_). The lower panel shows the distribution of the electron mean free path, which models a set of quasi-random pinning centres. The magnetic field distributions, which are formed in the course of a tunnelling current pulse (upper panel) and after switching off the pulse (lower panel) are shown for the parameter *α*=0.2 (**c**), 0.3 (**d**) and 0.5*T*_c_ (**e**). When the tunnelling current is on, local superconductivity is fully suppressed. The scale bar equals 4 μm.

The proposed scenario of vortex clustering is supported by the results of numerical simulations, based on the time-dependent Ginzburg–Landau (TDGL) formalism and the local heating model (Supplementary Fig. 10, Supplementary Note 6). The deviation of the local temperature *T* from its equilibrium value in the absence of tunnelling current, *T*_0_, is represented as *T*−*T*_0_=*αf*(*x*, *y*) (Fig. 4a), where the coefficient *α* is proportional to the power dissipated by the tunnelling current, while the spatial temperature distribution *f*(*x*, *y*) is determined by material and geometric parameters of the sample. Using this temperature distribution to model the effect of a tunnelling current pulse, the TDGL calculations are performed for a 10 × 10 μm^2^ superconductor film with random pinning centres (Fig. 4b). Based on the used local heating model and the material parameters, the quenching time 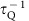
=−*T*^−1^∂*T*/∂*t*|_*T*=*T*_c__, corresponding to the thermal relaxation of the hot spot after switching off the tunnelling current, is estimated to lie within the range of 1–10 ps for the initial radii of the normal domain in the hot spot from 1 to 3 μm. These quenching-time values are comparable to the Ginzburg–Landau characteristic time in our sample, *t*_GL_(*T*_0_)=1 ps. The formation of vortex clusters, resulting from the simulated quenching process, and their stabilization due to vortex pinning typically occurs on the time scale ∼40–100 ps. The vortex cluster formation time, compared with the short quenching time, clearly places the quenching process in the KZ regime.

The simulation results are displayed in Fig. 4c–e for different values of the coefficient *α*. The local heating, caused by tunnelling current, leads to the formation of a normal-state region in the vicinity of the STM tip. The normal region can be considered as a big pinning site, which is capable to accommodate a relatively large magnetic flux. This pinning site attracts and traps vortices from the surrounding superconducting area, where the temperature is elevated and the mobility of vortices is increased due to their weaker interaction with pinning centres. The magnetic flux trapped within the normal region increases with increasing *α*. When the tunnelling current is switched off and the local temperature relaxes to *T*_0_, the trapped flux transforms into a compact vortex cluster, which is stabilized by the pinning centres. Similarly to the experiment, the calculated vortex pattern demonstrates the appearance of vortex-depleted regions around the vortex cluster, which become more evident when increasing the intensity of local heating and the cluster size.

### Manipulation of single-quantum vortices

**Figure 5:**
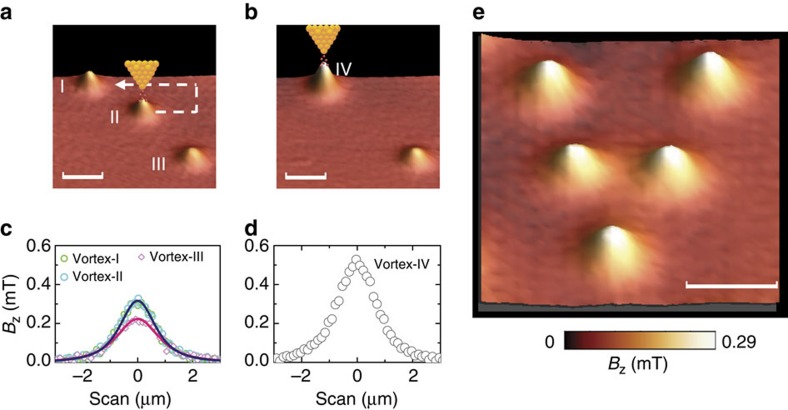
Manipulation of individual vortices with the STM tip. (**a**) SHPM image observed after FC from above *T*_c_ to *T*=4.2 K at *H*_0_=4.3 A m^−1^. Three single flux quantum vortices are observed at naturally formed pinning centres. (**b**) Image observed after moving vortex II with the STM tip along the arrow in **a**. Vortices I and II merged to form a vortex cluster IV. (**c**,**d**) Magnetic field profiles through the centres of vortices I, II, III and vortex cluster VI. The solid lines are a fit with the monopole model. (**e**) A pattern V short for vortex is arranged by using the STM tip. The scale bars equal 4 μm for all the images.

The obtained results suggest that local heating with an STM tip can provide an efficient tool to arrange vortices in superconductors. Finally, we demonstrate the ability of using the STM tip to manipulate individual vortices (Supplementary Movie). This is an important extension of the pioneering work of Eigler and Schweizer^16^ from manipulating single atoms to manipulating single-quantum vortices. To move a vortex, first, a tunnelling junction is established by approaching the STM tip to the surface of the sample at 1 μm away from the vortex centre. A hot spot is generated and the vortex is attracted to the location of the hot spot. Then the STM tip is retracted and moved to the next position. By repeating the above process, we are able to drag the vortex to any position in the superconductor. As shown in Fig. 5a, a single-quantum vortex (vortex II) can be moved along the path indicated by the dashed arrow to merge with Vortex I, forming a vortex cluster (IV in Fig. 5b). We are also able to drag the vortex cluster as one object to a new position and then detach the two vortices by increasing the temperature (Supplementary Fig. 11). By using the local heating method demonstrated above, a pattern V, short for vortex, is arranged from Φ_0_-vortices as shown in Fig. 5e. Compared with other techniques, such as magnetic force microscopy^6^, that are used to manipulate individual vortices, the important advantage of our technique is that no magnetic field or transport current is needed.

## Discussion

In conclusion, we have shown that a controlled heating effect generated by the tunnelling current can be used to locally suppress superconductivity, with this area playing the role of a pinning centre. We are able to tune the power of the nano heater simply by changing the bias voltage. Moreover, the tunnelling pulse applied through the STM tip enables dragging and manipulation of individual quantized vortices which may be used for the development of new fluxon-based devices. Our results demonstrate the utility of the local heating effect for characterizing and manipulating vortices in a superconductor. This new approach provides a lot of information which is of great interest for studying other systems, such as local and non-local phase transitions in superfluid, cosmology and many-particle systems.

## Methods

### Sample and experimental setup

The trilayer of Au/Ge/Pb was prepared in two steps. First, to ensure a relatively small *κ* of the sample, a 85 nm-thick Pb film was deposited on a SiO_2_/Si substrate using an ultra high vacuum (3 × 10^−8^ Torr) electron beam evaporator calibrated with a quartz monitor. The substrate was cooled to 77 K by liquid nitrogen to ensure the homogeneous growth of Pb. On top of Pb, a 10 nm thick Ge layer is deposited to protect the sample surface from oxidation and also to avoid any proximity effect between Pb and the subsequently deposited conductive layer^34^. Second, the sample was transferred into a sputtering machine, and a 35 nm thick Au layer was deposited to cover the whole sample, playing the role of a conducting layer for the tunnelling current of the STM tip. The thickness of Au yields a smooth surface with a roughness less than 0.2 nm. The critical temperature *T*_c_=7.25 K is determined using local ac susceptibility measurements. The pulse tunnelling current was applied by controlling the bias voltage between the STM tip (Au) of a commercial Hall probe shown in Fig. 1b and the sample, which is grounded. In our experiments, the tunnelling current is kept constant (∼0.5 nA) through a PID protocol control. The magnetic field distribution is recorded using the scanning Hall probe microscope from nanomagnetics in a lift-off mode (Supplementary Fig. 12, Supplementary Note 7). First, the Hall sensor approaches the sample under the control of a piezo until the tunnelling current is established. Then the whole sensor is retracted by 200 nm and the magnetic field distribution is mapped. In all the measurements, the magnetic field is applied perpendicularly to the sample surface. WSxM software is used to process all SHPM images^35^.

### TDGL simulation

For a superconductor with pinning centres, which originate from a local reduction of the mean free path 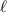, the TDGL equation for the order parameter 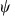, normalized to 1, can be written in the form^26^


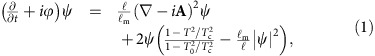


where 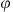 and **A** are the scalar and vector potentials, respectively, and *l*_m_ is the mean free path value outside the pinning centres. The relevant quantities are made dimensionless by expressing lengths in units of 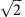
*ξ*(*T*_0_), time in units of*π*ℏ/[4*k*_B_(*T*_c_−*T*_0_)]=2*t*_GL_(*T*_0_), magnetic field in units of Φ_0_/[4*πξ*^2^(*T*_0_)]=*μ*_0_*H*_c2_(*T*_0_)/2, and scalar potential in units of 2*k*_B_(*T*_c_−*T*_0_)/(*πe*). Here, *μ*_0_ is the vacuum permeability, *t*_GL_ is the Ginzburg–Landau time, *H*_c2_ is the second critical field and *ξ* is the coherence length at *l*=*l*_m_.

The vector potential **A**, for which we choose the gauge **∇**·**A**=0, can be represented as **A**=**A**_0_+**A**_1_. **A**_0_ denotes the vector potential corresponding to the externally applied magnetic field **H**_0_, while **A**_1_ describes the magnetic field **H**_1_ induced by the currents **j**, which flow in the superconductor:


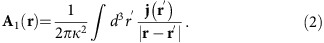


Here, *κ*=*λ*(*T*_0_)/*ξ*(*T*_0_) is the Ginzburg–Landau parameter and *λ* is the penetration depth at 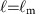. The current density is expressed in units of Φ_0_/[2
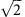
*πμ*_0_*λ*(*T*_0_)^2^*ξ*(*T*_0_)]=3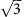/(2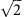)*j*_c_(*T*_0_) with *j*_c_, the critical (depairing) current density of a thin wire or film. Integration in equation (2) is performed over the volume of the superconductor.

In general, the total current density contains both the superconducting and normal components: **j**=**j**_s_+**j**_n_ with


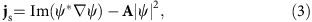



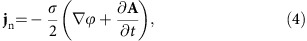


where *σ* is the normal-state conductivity, which is taken as *σ*=1/12 in our units^36^. The distribution of the scalar potential 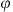 is determined from the condition


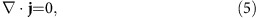


which reflects the continuity of currents in the superconductor. Both the *j*_n_ and 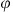 vanish when approaching (meta)stable states, which are of our main interest here.

Assuming that the thickness of the superconductor layer is sufficiently small, variations of the order parameter magnitude across the sample as well as currents in this direction can be neglected and equation (1) becomes effectively two-dimensional. This equation together with equations (2) and (5) are solved self-consistently following the numerical approach described in ref. 37.

### Data availability

All relevant data are available from the corresponding author.

## Additional information

**How to cite this article:** Ge, J.-Y. *et al*. Nanoscale assembly of superconducting vortices with scanning tunnelling microscope tip. *Nat. Commun.* **7,** 13880 doi: 10.1038/ncomms13880 (2016).

**Publisher's note:** Springer Nature remains neutral with regard to jurisdictional claims in published maps and institutional affiliations.

## Supplementary Material

Supplementary Information Supplementary Figures 1-12, Supplementary Noted 1-7 and Supplementary References
