# Supplementary material for: Nanoscale assembly of superconducting vortices with scanning tunnelling microscope tip
Source: Nat Commun. 2016 Dec 9;7:13880. doi: 10.1038/ncomms13880 (PMC5155158; doi:10.1038/ncomms13880)
Supplement: Supplementary Information — Supplementary Figures 1-12, Supplementary Noted 1-7 and Supplementary References [file ncomms13880-s1.pdf]

Supplementary Figures

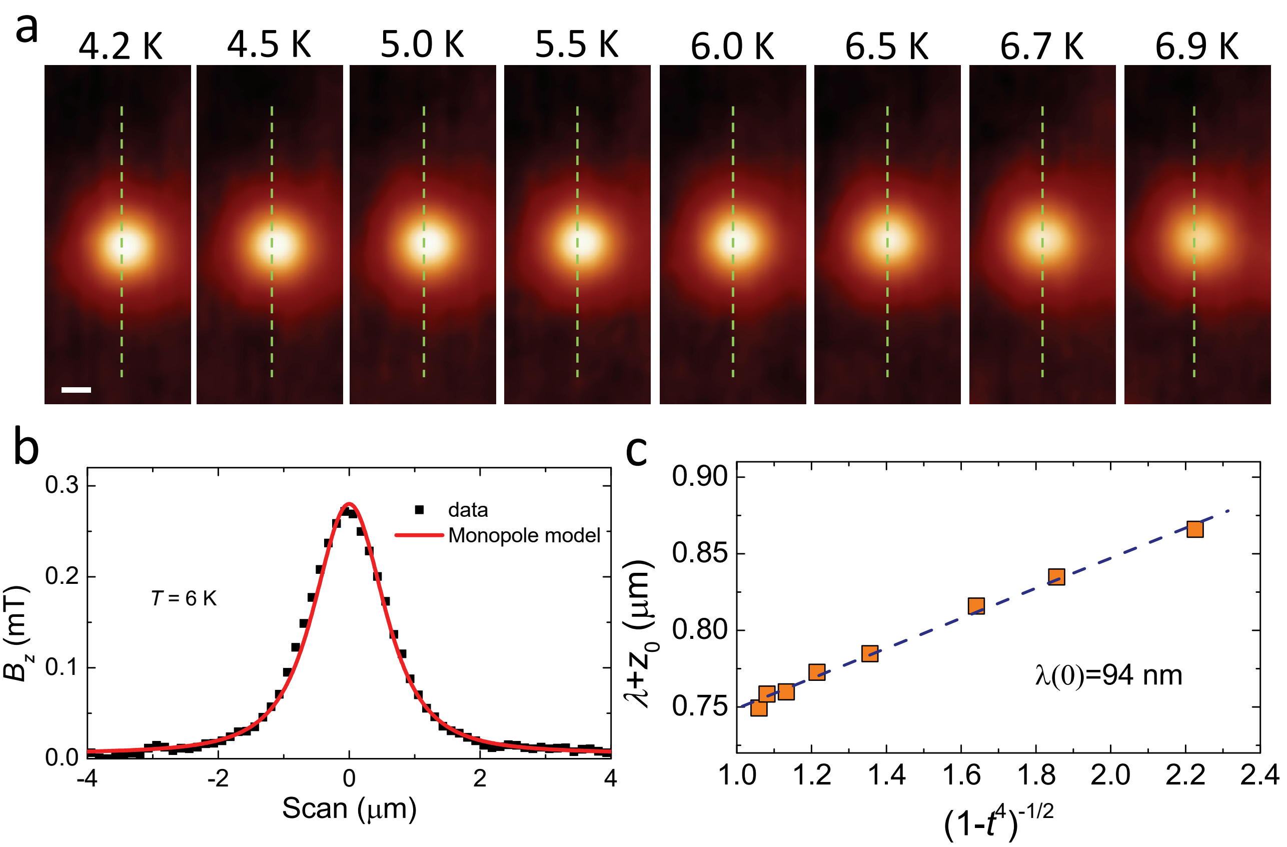

Supplementary Figure 1: Determination of the penetration depth. (a) SHPM images of a single quantum vortex observed at various temperatures as indicated. Scale bar equals 1 μm. (b) Field profile along the dashed line for the vortex at $T = 6$ K. Solid line is the monopole model fit. (c) $\lambda+z_0$ vs $(1-t^4)^{-1/2}$. Dashed line is the linear fit.

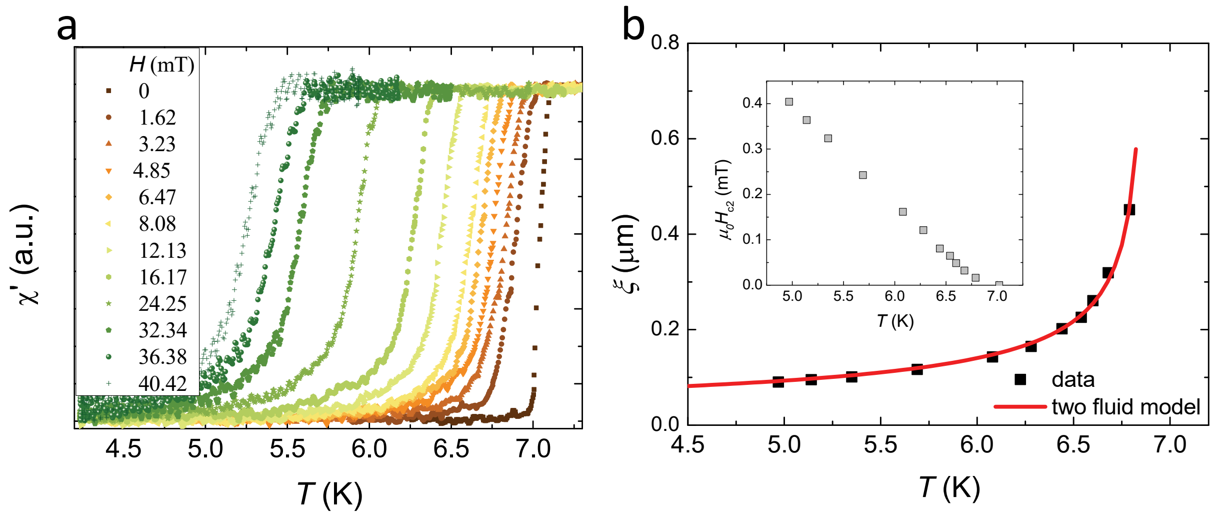

Supplementary Figure 2: Determination of the coherence length. (a) Temperature dependence of the ac susceptibility under various magnetic fields. (b) Temperature dependence of the coherence length. Solid line is the fit with the monopole model. Inset shows the H_{c2}-T phase diagram.

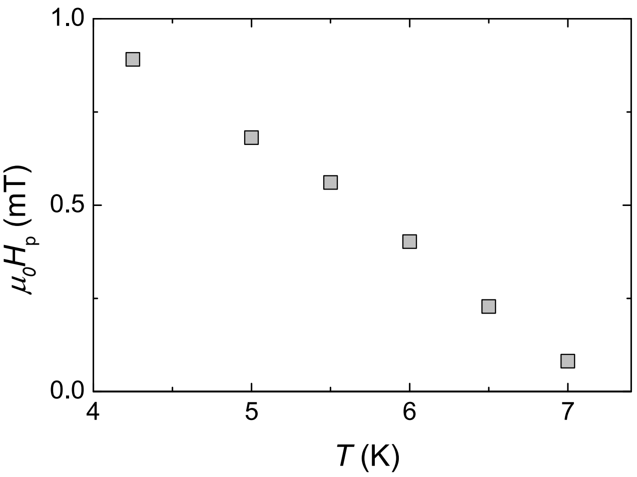

Supplementary Figure 3: Temperature dependence of the penetration field.

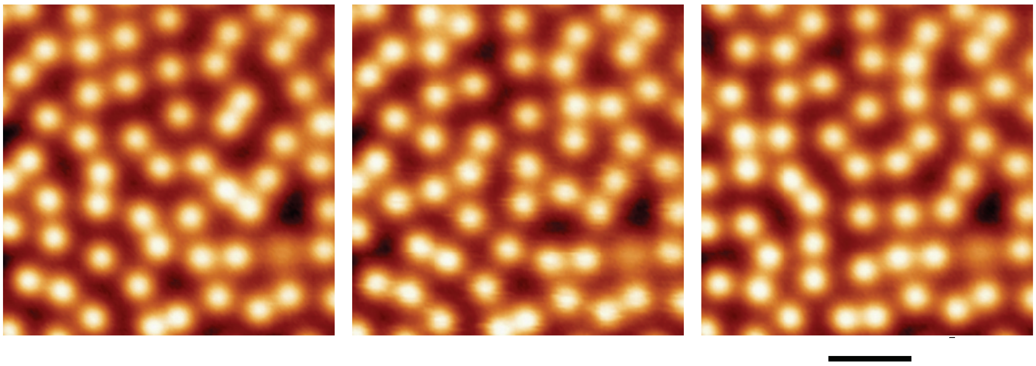

Supplementary Figure 4: Vortex patterns observed at the same area after performing a series of FC processes to 4.2 K with $\mu_0 H_0$ = 0.47 mT. Scale bar equals 4 μm.

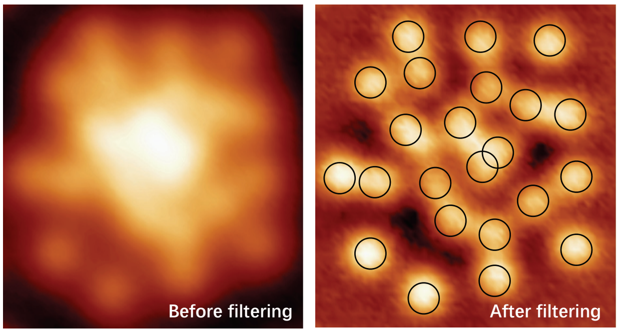

Supplementary Figure 5: Vortex cluster pattern before (left) and after (right) performing Fourier filtering.

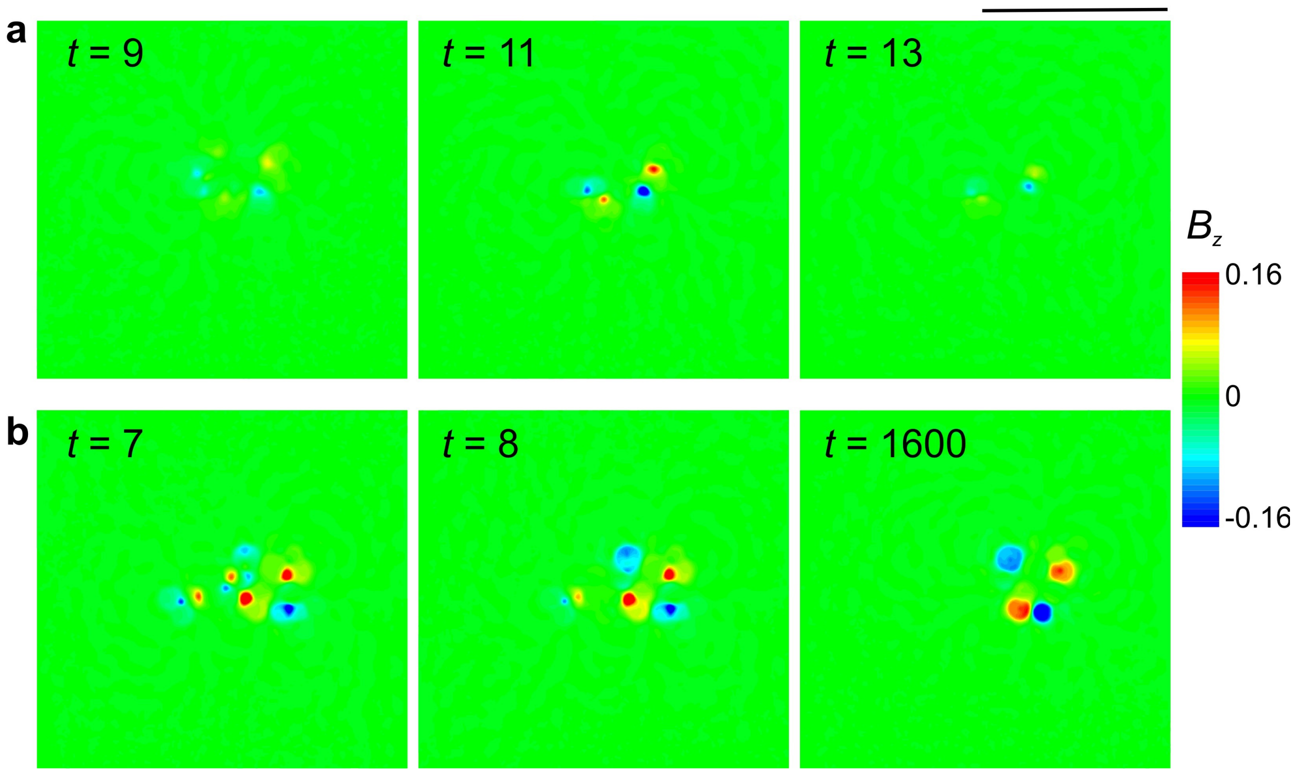

Supplementary Figure 6: Simulation of the KZ vortices. Calculated magnetic field distributions at the superconductor surface for $\alpha = 0.5T_c$ at $H_0 = 0$ and different time t, counted from the start of the quenching process. The quenching time, defined as $\tau_Q = -T^{-1}\partial T/\partial t|_{T=T_c}$, equals 5 (a) and 2 (b). For the sample parameters, indicated in the manuscript, our time unit approximately corresponds to 2 ps. The magnetic field unit is $\mu_0 H_{c2}/2$. The scale bar equals 5 μm.

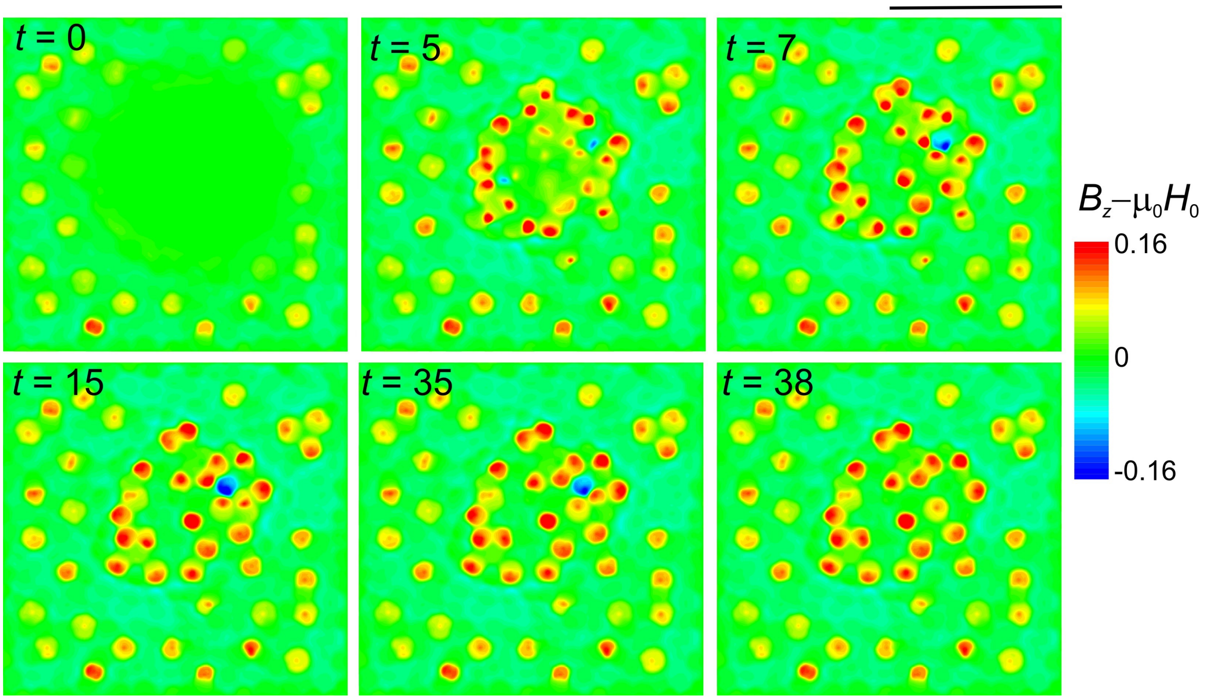

Supplementary Figure 7: KZ vortex-antivortex pairs generated in the presence of magnetic field. Calculated magnetic field distributions at the superconductor surface for $\alpha = 0.5T_c$ and quenching time $\tau_Q = 1$ at different time t, counted from the start of the quenching process. The scale bar equals 5 μm.

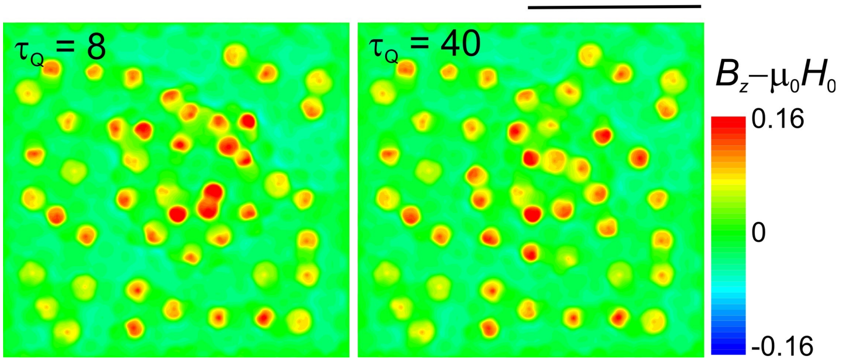

Supplementary Figure 8: Vortex cluster formation at different quenching time. Calculated magnetic field distributions at the superconductor surface, reached as a result of the quenching process, for $\alpha = 0.5T_c$ and different quenching time τ_Q. The scale bar equals 5 μm.

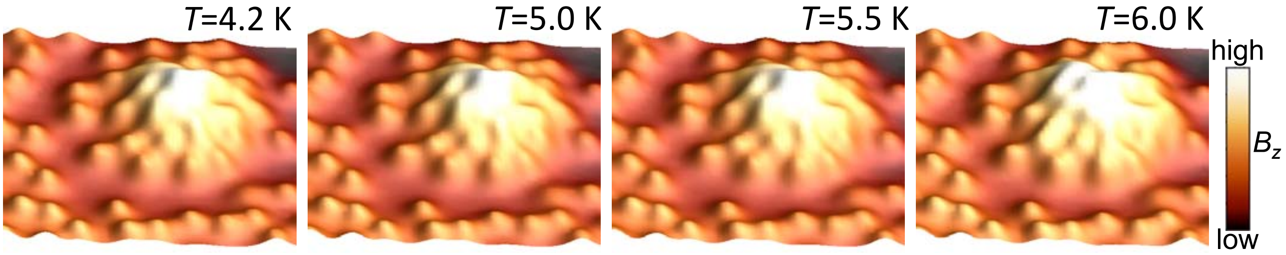

Supplementary Figure 9: **Vortex cluster evolution in low temperature range.** At low temperatures, the interaction between pinning centers and vortices dominate. As a result, the vortex cluster retains the same geometry up to $T = 6.0$ K. Only at higher temperatures, the vortex-vortex repulsion overcomes the attractive interactions between pinning centers and vortices, then the vortex cluster starts to decompose as illustrated in Fig. 3 of the manuscript. The scale bar equals 4 μm.

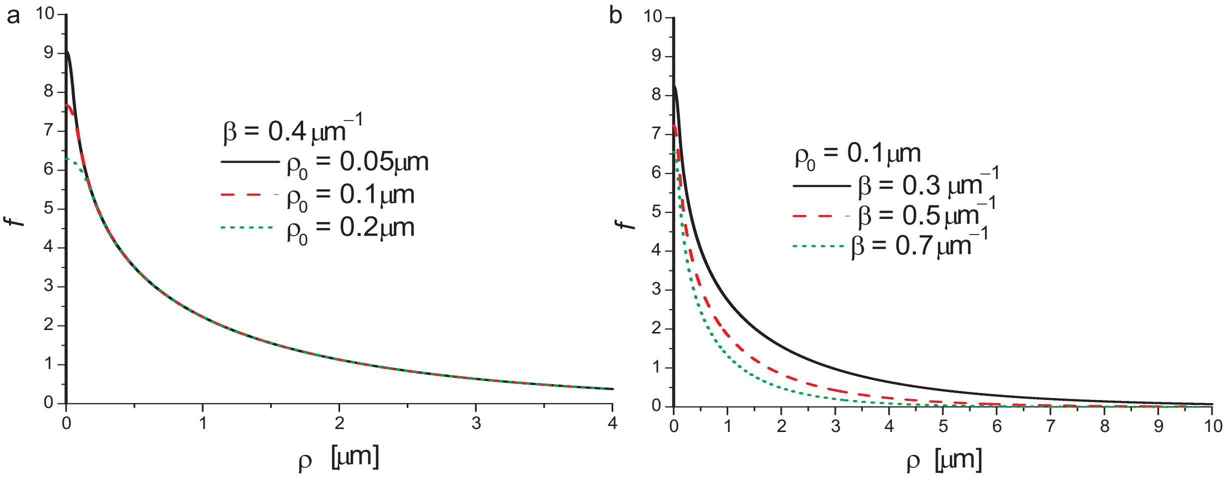

Supplementary Figure 10: Function $f(\rho)$, which determines the shape of the temperature distribution in the superconductor layer, at $\beta = 0.4\ \mu m^{-1}$ and different ρ_0 (a) and at $\rho_0 = 100$ nm and different β (b).

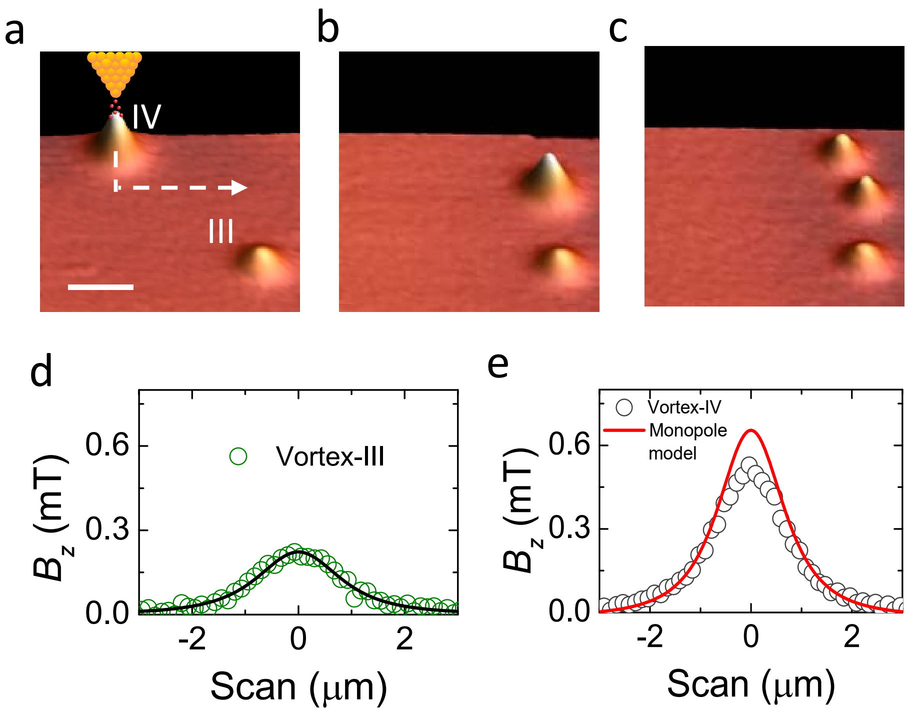

Supplementary Figure 11: **Manipulation and detachment of a vortex cluster.** (a) The same vortex pattern as shown in Fig. 5b of the manuscript. The vortex cluster IV carries two Φ_0-vortices. By using the STM tip, we are able to drag the vortex cluster as one object to a new position as shown in (b). (c) SHPM image observed by warming the vortex pattern in (b) to 6.8 K and then decreasing the temperature to 4.2 K. The vortex cluster is decomposed into two Φ_0-vortices. The scale bar equals 4 μm. The magnetic field profiles through the center of vortex III (d) and the cluster IV (e). The solid lines are the fit of the monopole model with the magnetic flux fixed at (d) Φ_0 (e) $2\Phi_0$ (a two-quanta vortex). Clearly, the vortex cluster cannot be well fitted with a multi-quanta vortex. This suggests that IV is composed of two Φ_0-vortices, which are placed close to each other.

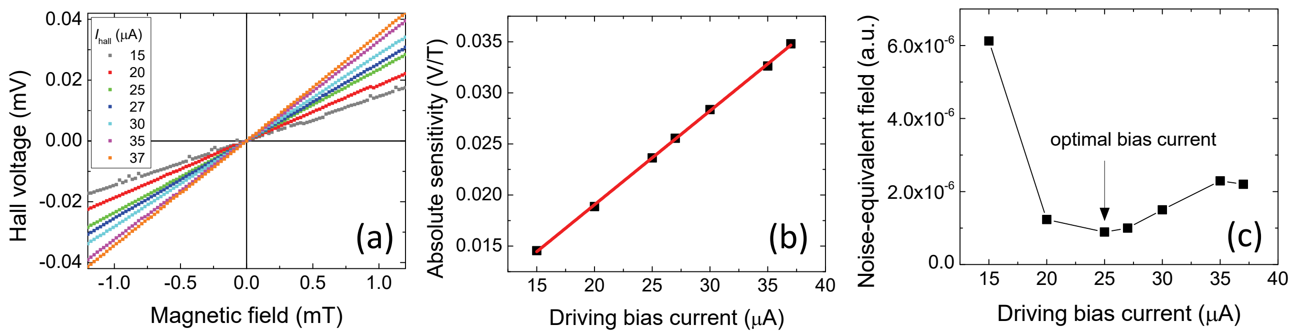

Supplementary Figure 12: Hall sensor calibration. (a) Hall voltage as a function of magnetic field for different driving bias current. (b) Absolute sensitivity as a function of driving current. (c) Noise-equivalent field as a function of different driving bias current.

Supplementary Notes

Supplementary Note 1: Determination of the penetration depth.

The penetration depth is determined by using the monopole model to fit the vortex profile observed at various temperatures. Supplementary Figure 1a shows the SHPM images observed at various temperatures indicated above each image. By fitting the magnetic field profiles along the dashed line, e.g. for $T = 6$ K shown in Supplementary Figure 1b, we can get the value of $\lambda+z_0$ at different temperatures, where λ is the penetration depth and z_0 is the distance between the two-dimensional electron gas (TDEG) of our Hall cross and the sample surface (this distance is constant). According to the two-fluid model, the penetration depth follows a linear dependence on $(1-t^4)^{-1/2}$ with $t = T/T_c$. As shown in Supplementary Figure 1c, from the slope of the linear fitting, we can get the penetration depth at zero temperature.

Supplementary Note 2: Determination of the coherence length and penetration field

To determine the coherence length, we measured the temperature dependence of the in-phase ac susceptibility at different magnetic fields, as shown in Supplementary Figure 2a below. The temperature dependence of H_{c2} (inset of Supplementary Figure 2b) can be deduced from the χ'-T curve. The coherence length is calculated with the relation $H_{c2}(T) = \Phi/2\pi\xi(T)$, as shown in Supplementary Figure 2b. The data can be well fitted by the two-fluid model (solid line), yielding $\xi(0) = 52$ nm.

We have measured the penetration field at various temperatures using the criterion that the first vortex is seen to penetrate into the sample. The results are shown in Supplementary Figure 3. Here the critical field should be regarded as the penetration field H_p. Due to the sample geometry which leads to a large demagnetization effect, the real lower critical field should be considerably larger than the penetration field.

Supplementary Note 3: Determination of the number of vortices in a vortex cluster

To determine the number of trapped vortices in the cluster, two different ways are used:

i) The applied magnetic field induces the nucleation of vortices in a superconducting area with the number N that can be determined as $N = HS/\Phi_0$, where H is the applied magnetic field, S is the superconducting area and Φ_0 is the flux quantum. For the applied field value $\mu_0 H_0 = 0.47$ mT used in Fig. 2a of the manuscript, $N = 58$ single quantum vortices are expected in our scanning area of 16x16 μm^2. The Supplementary Figure 4 shows three vortex patterns at the same area after performing a series of field cooling processes at $\mu_0 H_0 = 0.47$ mT. In each pattern the observed

vortex number is N = 58±2, that is consistent with the expected number. The uncertainty is due to the vortices cut by the edges. To determine the number of vortices trapped in the cluster in Fig. 2a, we simply count the number (N_1) of vortices outside the cluster. Then the number of vortices trapped can be determined as N-N_1.

ii) The number of trapped vortices inside the cluster can also be roughly checked by first performing Fourier transform of the cluster pattern and then analyze the high frequency signal from the image (remove low frequency background). For example, Supplementary Figure 5 shows the vortex cluster pattern (corresponding to the cluster at V_{bias}=0.4 V in Fig. 2a) before (left) and after (right) filtering. From the right hand side image, we count 23 vortices in the cluster as indicated by the circles, which is fully consistent with the number (23) found by using method i). The results also suggest that there is no multi-quanta vortex in the cluster.

Supplementary Note 4: Monopole model

In the limit of $(r^2+z_0^2)>>\lambda^2$, where $\boldsymbol{r}=(x,y)$ is the distance from the vortex center, λ is the penetration depth, z_0 is the distance from the sample surface to the two-dimensional electron gas of the Hall cross, the magnetic field profile of a vortex can be closely approximated by the monopole model with the following expression [1-3]:

$$B_z(r)=\frac{\Phi}{2\pi}\frac{\lambda+z_0}{\left[r^2+(\lambda+z_0)^2\right]^{3/2}} \quad (1)$$

Here, $B_z(r)$ is the magnetic field perpendicular to the sample surface and Φ is the total flux carried by a vortex. According to Ref. [4], the accuracy of the model can be enhanced by averaging over an area representative of the Hall probe active area to account for the convolution of the field over the probe. The integration of the equation above over a square active area of size s and divided by the area s^2 gives the following result:

$$B_z(x,y)=\frac{\Phi}{2\pi s^2}\int_{y-\frac{s}{2}}^{y+\frac{s}{2}}dy\int_{x-\frac{s}{2}}^{x+\frac{s}{2}}dx\frac{\lambda+z_0}{\left[x^2+y^2+(\lambda+z_0)^2\right]^{3/2}} \quad (2)$$

For our SHPM, a Hall probe with an active area of s^2=0.4x0.4 μm^2 is used.

From supplementary Equation (2), it is clear that a doubly quantized vortex would double the magnetic field at each point. However, if the distance between the two adjacent pinning centers is much smaller than the spacial resolution of our scanning technique, it is rather difficult to distinguish between a giant $2\Phi_0$ vortex and a vortex $\Phi_0 + \Phi_0$ cluster. As a result, the observed field distribution of a cluster with two Φ_0-vortices could still be doubled as compared with a single quantum vortex, as is the case shown in Fig. 2b of the manuscript.

Supplementary Note 5: Theoretical simulations of the Kibble-Zurek phase transition by the local heating effect

The Kibble-Zurek (KZ) scenario developed for homogeneous systems is modified in the case of a spatial inhomogeneity of the quench, resulting in movement of the temperature front [5-7]. As shown in Ref. [8], for a quenched normal spot in a superconductor subjected to an external magnetic field, such a modified scenario leads to the formation of vortex rings, which may qualitatively resemble vortex chains predicted for inhomogeneous quenching of normal fluid regions in superfluids in the presence of external superflow [6,7].

Based on the used local heating model and the material parameters, the quenching time, corresponding to the thermal relaxation of the hot spot after switching off the tunneling current, is estimated to lie within the range of 1 to 10 ps for the initial radii of the normal domain in the hot spot from 1 to 3 μm. These quenching-time values are comparable to the Ginzburg-Landau characteristic time in our sample, $t_{GL}(T_0) \approx 1$ ps. The formation of vortex clusters, resulting from the simulated quenching process, and their stabilization due to vortex pinning typically occur on the time scale ~40 to 100 ps. In this note, we consider two situations: i) zero flux state ii) finite flux state.

i) Zero flux state. The local hot spots, considered in our manuscript, are well separated from the edges of the superconducting samples so that the total vorticity number in the corresponding regions is always preserved. Therefore, the KZ topological defects can appear there only in the form of vortex-antivortex pairs, as illustrated in Supplementary Figure 6, which shows the simulated results of the hot spot quenching for the vortex-free state in the absence of an applied magnetic field. As seen from Supplementary Figure 6, due to a strong vortex-antivortex attraction most of the nucleated pairs rapidly recombine without having chance to be fully developed and spatially separated. Only in one out of the ten performed simulation runs, two of the nucleated vortex pairs have appeared to be sufficiently strongly pinned and hence long-living (see Supplementary Figure 6b). Our attempts to reveal (meta)stable vortex-antivortex pairs in the experimental samples at zero applied magnetic field failed. We attribute this fact to an insufficiently strong vortex pinning in these samples.

ii) Finite flux state. Our simulations further demonstrate that the nucleation of KZ vortex pairs in the course of the hot spot quench occurs also in the mixed state at relatively strong magnetic fields, when the normal domain in the hot spot confines a large magnetic flux (see Supplementary Figure 7). In this situation, however, a high density of vortices, released by the quenching hot spot, leads to a much larger annihilation probability for an antivortex as compared to the afore-described case, where the total vorticity in the hot spot is zero. This explains the absence of frozen antivortices both in the simulation results and experimental data related to vortex-cluster formation.

Our simulations further show that an increase of the quenching time as compared to the values, corresponding to the formation of KZ topological defects, results in washed out patterns of the formed vortex cluster, which approach the vortex configurations typical for an adiabatic cooling

(see Supplementary Figure 8). In view of this, the observation of well-defined vortex clusters in the performed measurements can be considered as an indication that the experimental conditions correspond to a realization of the KZ scenario.

Supplementary Note 6: Local heating model

In the experimental sample the relatively thin Au/Ge/Pb trilayer is separated from the thick Si substrate by a SiO_2 layer. At the relevant temperatures, thin amorphous SiO_2 films are characterized by a remarkably small thermal conductivity k [9], which strongly decreases with decreasing the film thickness d [10]. For the experimentally relevant SiO_2 layer thickness (d=300 nm) the thermal conductance per unit area in the z direction, $K_z=k/d$, can be estimated as $K_z \approx 60$ W/(cm^{2}K). Just the low thermal conductance of the SiO_2 layer mainly determines the efficiency of the heat removal from the Au/Ge/Pb trilayer. Due to much higher thermal conductivities of the Au [11], Ge [12] and Pb [13] layers and their relatively small thicknesses, the estimated thermal conductance of the Au/Ge/Pb trilayer in the z direction exceeds that of the SiO_2 layer by two orders of magnitude. In view of this, the temperature gradient across the Au/Ge/Pb trilayer is rather negligible and we can calculate the temperature distribution in this trilayer within a 2D model, similar to that used in Refs. [14,15]. Assuming that this distribution is axially symmetric with respect to the STM-tip position the heat equation can be written as

$$CD\frac{\partial u}{\partial t} = \bar{k}D\frac{\partial^2 u}{\partial \rho^2} + \frac{\bar{k}D}{\rho}\frac{\partial u}{\partial \rho} + q - K_z u. \tag{3}$$

Here, ρ is the polar radius, $u = T - T_0$ describes the deviation of the local temperature T in the Au/Ge/Pb trilayer from the equilibrium temperature T_0 in the absence of tunnelling current, C is the effective volumetric heat capacity of this trilayer, D is its total thickness, $\bar{\kappa}$ is the average in-plane thermal conductivity of the trilayer, and q is the surface density of the power dissipated by the tunnelling current. The last term in the right hand side of Supplementary Equation (3) describes the heat removal from the trilayer through the SiO_2 layer. For simplicity, we take

$$q = \begin{cases} q_0, & \rho \le \rho_0, \\ 0, & \rho > \rho_0, \end{cases} \tag{4}$$

where $q_0 = P/(\pi \rho_0{}^2)$. Here, P is the power dissipated by the tunneling current and ρ_0 is the radius of the region, where this dissipation occurs. When analyzing the effect of a tunneling current pulse on vortex distributions, the precise value of ρ_0 and the detailed form of $q(\rho)$ at $\rho \le \rho_0$ are not really important, provided that ρ_0 is much smaller than the size of the normal region, induced by local heating. In our simulations below we use ρ_0 = 100 nm.

The temperature profile, which affects the order- parameter distribution in the superconductor layer, corresponds to the steady-state solution of Supplementary Equation (3). This solution can be written in terms of the modified Bessel functions $I_\nu(x)$ and $K_\nu(x)$ [16]:

$$T(\rho) - T_0 = \alpha f(\rho) \quad (5)$$

$$f(\rho) = \frac{4}{\beta^2\rho_0^2}\begin{cases} 1 - \beta\rho_0 K_1(\beta\rho_0) I_0(\beta\rho), & \rho \le \rho_0, \\ \beta\rho_0 I_1(\beta\rho_0) K_0(\beta\rho), & \rho > \rho_0. \end{cases} \quad (6)$$

Here the coefficient $\alpha = P/(4\pi\bar{\kappa}D)$ is proportional to the power dissipated by the tunneling current, while the parameter $\beta = [K_Z/(\bar{\kappa}D)]^{1/2}$ is estimated to be 0.4 μm^{-1} for the sample under consideration. The function $f(\rho)$ at various values of β and ρ_0 is shown in Supplementary Figure 10.

Supplementary Note 7: Calibration of the Hall probe

The used Hall probe is calibrated before the measurements. The Hall voltage V_{Hall} was measured as a function of the perpendicular magnetic field H for different driving currents I. Supplementary Figure 12a shows the $V_{Hall}(H)$ curves for the used Hall sensor. Linear fits to the $V_{Hall}(H)$ curves yield the absolute sensitivity S_A=dV_{Hall}/dH. As shown in Supplementary Figure 12b, S_A is a linear function of the driving current. From a fit to the linear portion of the $S_A(I)$ curve, the current sensitivity S_I=dS_A/dI is obtained. From each $V_{Hall}(H)$ curve, the standard deviation from the linear fit yields the Hall voltage noise δV_{Hall}. With these values, the noise-equivalent magnetic field $B_{noise}=\delta V_{Hall}/IS_I$ was determined for each bias current value I. Supplementary Figure 12c shows the noise-equivalent value, from which we can observe the optimal bias current (25 μA) for our Hall cross with the lowest noise level. The absolute sensitivity at I = 25 μA is 2.363×10^{-2} VT^{-1}. Using the absolute sensitivity, the measured Hall voltage is directly converted to magnetic field by the SPM software.

Supplementary references

[1] Pearl, J. J. Structure of superconductive vortices near a metal-air interface. *J. Appl. Phys.* **37**, 4139-4141 (1966) .

[2] Chang, A. M. *et al*. Scanning Hall probe microscopy. *Appl. Phys. Lett.* **61**, 1974-1976 (1992) .

[3] Wynn, J. C. *et al.* Limits on spin-charge separation from *h*/2*e* fluxoids in very underdoped YBa2Cu3O6+*x*. *Phys. Rev. Lett.* **87**, 197002 (2001) .

[4] Guikema, J. W. Scanning Hall Probe Microscopy of Magnetic Vortices in Very Underdoped yttrium-barium-copper-oxide PhD thesis Stanford Univ. (2004) .

[5] Kopnin, N. B. & Thuneberg, E. V. Time-Dependent Ginzburg–Landau Analysis of Inhomogeneous Normal-Superfluid Transitions. *Phys. Rev. Lett.* **83**, 116-119 (1999).

[6] Aranson, I. S., Kopnin, N. B. & Vinokur, V. M. Nucleation of Vortices by Rapid Thermal Quench. *Phys. Rev. Lett.* **83**, 2600-2603 (1999).

[7] Volovik, G. E. Defect formation in inhomogeneous second-order phase transition: theory and experiment. *Physica B* **280**, 122-127 (2000).

[8] Shapiro, I., Pechenik, E. & Shapiro, Y. Recovery of superconductivity in a quenched mesoscopic domain. *Phys. Rev. B* **63**, 184520 (2001).

[9] Zeller, R. C. and Pohl, R. O. Thermal Conductivity and Specific Heat of Noncrystalline Solids *Phys. Rev. B* **4**, 2029-2041 (1971).

[10] Goodson, K. E., Flik, M. I., Su, L. T., Antoniadis, D. A. Prediction and Measurement of the Thermal Conductivity of Amorphous Dielectric Layers. *J. Heat Transfer* **116**, 317-324 (1994).

[11] Langer, G., Hartmann, J., and Reichling, M. Thermal conductivity of thin metallic films measured by photothermal profile analysis. *Rev. Sci. Instrum*. **68**, 1510-1513 (1997).

[12] Nath, P. and Chopra, K. L. Thermal conductivity of amorphous and crystalline Ge and GeTe films. *Phys. Rev. B* **10**, 3412-2418 (1974).

[13] Zink, B. L., Revaz, B., Cherry, J. J., and Hellman, F. Measurement of thermal conductivity of thin films with a Si-N membrane-based microcalorimeter. *Rev. Sci. Instrum*. **76**, 024901 (2005).

[14] Gurevich, A.V. & Mints, R. G. Self heating in normal metals and superconductors. *Rev. Mod. Phys.* **59**, 941-999 (1987).

[15] Vodolazov, D. Y., Peeters, F. M., Morelle, M. & Moshchalkov, V. V. Masking effect of heat dissipation on the current-voltage characteristics of a mesoscopic superconducting sample with leads. *Phys. Rev. B* **71**, 184502 (2005).

[16] Abramowitz, M. and Stegun, I. A. Handbook of Mathematical Functions with Formulas, Graphs, and Mathematical Tables (New York: Dover, 1972).
